# Modelling of land nutrient cycles: recent progress and future development

**DOI:** 10.12703/r/10-53

**Published:** 2021-06-02

**Authors:** Ying-Ping Wang, Daniel S Goll

**Affiliations:** 1 CSIRO Oceans and Atmosphere, PMB 1, Aspendale Victoria 3195, Australia; 2 Université Paris Saclay, CEA-CNRS-UVSQ, LSCE/IPSL, Gif sur Yvette, France

**Keywords:** Nutrient cycle, global modelling, nitrogen, phosphorus

## Abstract

While widespread imitation of the productivity of the land biosphere by nutrients, like nitrogen and phosphorus, was demonstrated many decades ago, representation of nutrient cycles in global land models has been relatively recent. Over the last three years, significant progress has been made in understanding some of the key processes and their representation in global land models. They include the significance of plant–microbial interaction in affecting nutrient cycles, inorganic soil phosphorus transformation, and nitrogen release from rocks. As a result, our understanding of the linkages among geology, biology, and climate controlling nutrient cycles is improving. However, progress in modelling nutrient cycles at a global scale is still confronted with large uncertainties in representing key processes owing to lack of data at the relevant scales for evaluating coupled carbon and nutrient cycles. Here we recommend two approaches to advance modelling of land nutrient cycles: the application of machine learning techniques to bridge the gap between global modelling and scattered site-level information and the use of optimality principles to identify key mechanisms driving spatial and temporal patterns of nutrients.

## Introduction

The land biosphere has taken up about one-quarter of the cumulative anthropogenic CO_2_ emissions since the start of the industrial revolution, thereby reducing the amount of anthropogenic CO_2_ emissions that would otherwise remain in the atmosphere. Together with the ocean carbon sink, this carbon sink over land is currently slowing down anthropogenic global warming. To what degree this land carbon sink will continue into the future remains highly uncertain. A major source of uncertainty is to what extent the strength of the land carbon sink is controlled by soil nutrients^1^.

Since the 1990s, climate models used to predict future climate change have been extended to include biogeochemical processes, such as carbon cycling. Different from climate models, these earth system models project future climate change by accounting for the feedbacks between the changes in physical climate and the carbon cycle^2^. While none of the earth system models used for future climate projections in the fourth assessment report of the International Panel on Climate Change (IPCC) included nutrient cycles, five of the 11 earth system models in the forthcoming sixth IPCC assessment report have an explicit representation of nitrogen cycles and another model includes both nitrogen and phosphorus cycles^3^. In addition to earth system models, nutrient cycles have been included in more than 10 land biogeochemistry models^4^. However, while most models agree on a limiting effect of nutrients on the response of carbon uptake to increasing CO_2_ and climate change, the onset, strength, and evolution of nutrient limitation vary significantly among models^2,4^.

The development of land biogeochemical models is often confronted with a lack of (even basic) information needed to parameterize critical processes, hampering our quantitative understanding of many key processes and their interactions. While these models are based on a set of working hypotheses, evaluation, improvement, and further development of these models are needed to ensure that they keep pace with the ever-evolving knowledge in theory and increasing availability of observation-based data.

In this review, we summarize recent (over the last three years) advances in understanding of some of the key processes governing land nutrient cycles on scales relevant for the earth system. We focus on those advances that can inform future model developments to improve our capabilities to project future land carbon balance and in turn its effect on climate change. Finally, we identify some of the key gaps and present an outlook for the future development within the next five years.

## The cycles of nitrogen and phosphorus

At present, the only nutrient cycles that have been included in global land biogeochemical models are those for nitrogen and phosphorus, and their representations vary widely among models. Earlier approaches to include nutrient cycles relied on the use of prescribed carbon to nutrient ratios of organic matter pools, i.e. through fixed stoichiometric ratios, to couple nutrient cycles to the carbon cycles. Flexible stoichiometry is now used in most land models to accommodate the widely observed variations in carbon to nutrient ratios. Most but not all of the known major processes are included in current land nutrient models.

There are key differences between nitrogen and phosphorus cycles that theoretically lead to their distinct effects on response of the land biosphere to warming, increasing atmospheric CO_2_, and land use change.

**Figure 1.**
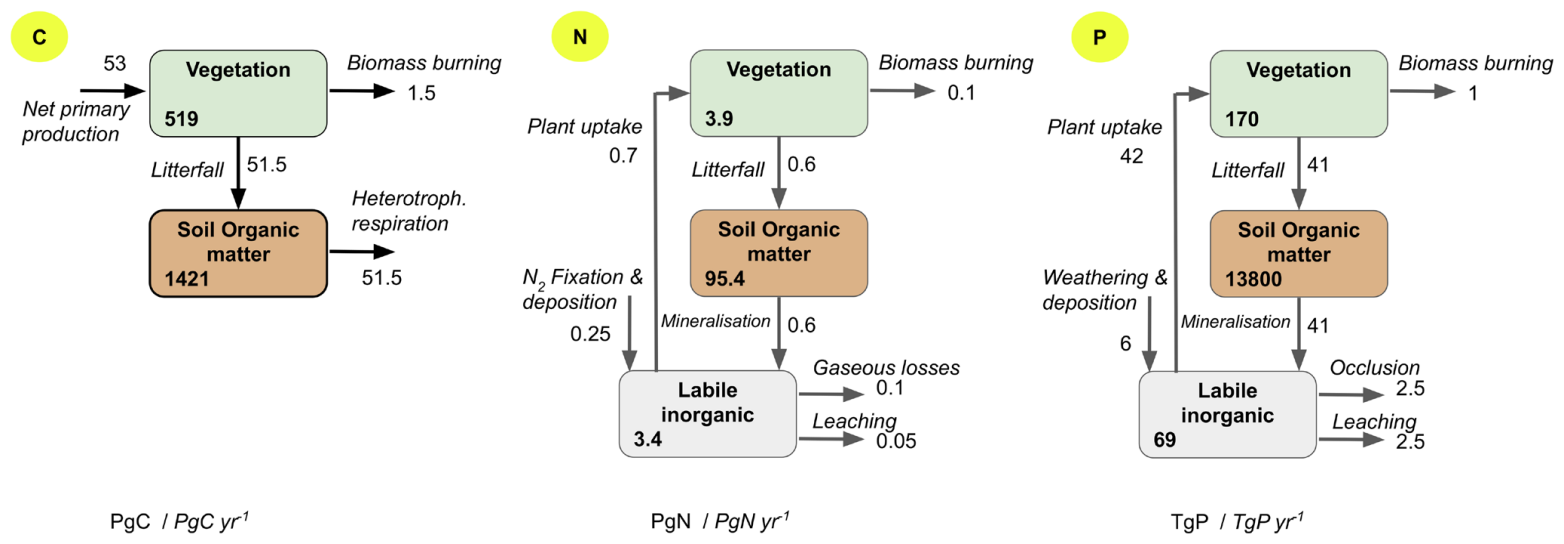
Cycles of carbon (C), nitrogen (N), or phosphorus (P) in vegetation biomass (Veg) and soil organic matter (SOM) or inorganic soil N or labile soil P on land as estimated by a model-data fusion framework^5^. The considered fluxes (*in italic*) and stocks (**in bold**) correspond to the ones commonly represented in most land biogeochemical models.

The terrestrial nitrogen cycle as shown in Figure 1 has two major inputs: biological nitrogen fixation and atmospheric deposition. A recent study^6^ quantified another potentially significant nitrogen input of 0.02 to 0.03 PgN/year from rock-weathering. These inputs together provide about 0.28 PgN/year to the land biosphere at present, or about 40% of the plant nitrogen uptake globally (Figure 1). These large inputs are balanced by comparable losses from soil and vegetation through gaseous emissions, leaching of dissolved nitrogen, and losses of particulate matter by water and wind erosion as well as by fires (Figure 1). Within ecosystems, nitrogen is recycled: plants take up inorganic nitrogen (nitrate or ammonium) and amino acids from soils and return nitrogen in organic form to the soil through litter fall and root exudates at a rate about 0.6 PgN/year. The organic nitrogen in soil or litter is mineralized by soil microbes, making it available again for plant uptake. In most terrestrial ecosystems, more than 95% of total soil nitrogen is in organic form.

Different from the nitrogen cycle, inputs and losses of phosphorus are small compared to the phosphorus pool sizes of ecosystems (Figure 1). Natural inputs include rock weathering and atmospheric deposition. Outputs are dominated by particulate losses from soil erosion, which are currently not represented in global land models but are estimated to be up to one order of magnitude larger than loss by leaching of dissolved phosphorus at a global scale^7^. Most of the annual inorganic phosphorus requirement of plants and soil biota is met through recycling from organic matter and desorption of organic and inorganic phosphorus in soil. Soil organic phosphorus is mineralized by soil microbes (biological mineralization) or through phosphatase enzymes or organic acids released to the soil by plants and microbes (biochemical mineralization). About 20 to 65% of soil phosphorus is inorganic, of which only a small fraction (<5%) is labile and biologically available. It has to be noted that some fraction of stabilized inorganic soil phosphorus is not represented in global land models, as that fraction is considered to be unavailable for biological uptake on the timescales of interest (less than a few centuries).

The relative concentration of carbon and nutrients (stoichiometry) in biomass can be quite variable depending on plant tissue type, plant species, leaf age, local climate, and soil conditions. Typically, leaf carbon to nitrogen is about 25 to 40 gC/gN for broadleaves and 40 to 80 gC/gN for needle leaves. Carbon to nitrogen ratio in woody tissue or coarse roots is much higher than leaf or fine root, varying from 150 to >1,000 gC/gN^5^. Leaf nitrogen to phosphorus ratio was observed to decrease with an increase in latitude, varying from >40 gN/gP in evergreen tropical forest to <20 gN/gP in the northern tundra, suggesting a decrease in phosphorus and an increase in nitrogen limitation from low to high latitudes.

## Recent advances in modelling nitrogen and phosphorus cycles

One of the greatest challenges in developing global land nutrient models is the lack of a (quantitative) understanding of some key processes that govern gains, losses, and recycling of nutrients at the ecosystem scale. While gains and losses of nutrients control the amount of nutrients, the internal recycling of nutrients within land ecosystems is a major control on nutrient availability. These three aspects have been shown to be critical for nutrient controls on land carbon uptake in theoretical studies^8^, and the representation of underlying processes remains rudimentary in most global land models. Despite these challenges, significant progress has been made in several key areas and is described in the following sections.

### Nutrient inputs

Biological nitrogen fixation (BNF) is presumably the largest natural nitrogen input to the land biosphere (about 60 to 110 TgN/year), and its response to increasing atmospheric CO_2_ and climate change will likely have a significant impact on the carbon balance of natural ecosystems^9^. However, BNF is only rudimentarily represented in land nutrient models. BNF is usually prescribed as a time-invariant model input or modelled using empirical relationships with ecosystem productivity or drivers thereof in global land models. This approach may underestimate biological nitrogen fixation rate in the future. As shown by Peng *et al*.^9^, the commonly used empirical approaches underestimate the increase in BNF, and thus underestimate future carbon uptake, particularly for evergreen needleleaf forests under future climate conditions and higher CO_2_ concentration. Given the importance of BNF on global land carbon uptake, significant research and progress are anticipated in BNF on land within the next few years through data synthesis and theoretical modelling^10^.

Nitrogen input from rock-weathering might have been overlooked as a major source for nitrogen. The pioneering work by Houlton, Morford, and Dahlgren^6^ estimated a substantial global source from rock-weathering of 19 to 31 TgN/year, which is about 10% of the total nitrogen inputs estimated by model-data fusion (Figure 1). If proven true, this would somewhat alter the spatial pattern of nitrogen scarcity and the evolution of the nitrogen cycle in global models due to environmental drivers, which differ from the ones of BNF.

### Nutrient recycling

***Nitrogen*.** As plants largely take up nutrients from the soil primarily in inorganic forms, recycling of nutrients from dead organic matter by microbe-mediated mineralization is critical for plant growth and nutrient cycling. Nutrient mineralization rate is usually modelled to vary with the amount and quality of substrate and environmental conditions, whereas the role of soil microbes has been largely ignored, primarily because of their minor biomass (a few percent of total soil organic carbon) and short turnover time (days). However, recent studies suggest that microbial dynamics control spatial variation and residence time of soil carbon^11,12^. So far, only a few models have included soil microbial processes explicitly^13,14^, one of which resolves dynamics of different soil enzymes and functioning of different classes of decomposers (bacteria, fungi, and macrofauna)^15^. By explicitly representing different classes of soil heterotrophic organisms, these models can simulate shifts in decomposer community composition and in the efficiency at which microbes convert substrate into microbial biomass, which are shown to affect nutrient recycling and land carbon balance under global changes in response to nitrogen addition, warming, and elevated CO_2_^15,16^.

Nutrients are taken up by plants through direct root uptake or by symbioses with root-associated fungi (mycorrhizae), which enhance nutrient uptake from soils with low available nutrients. Mycorrhizae mine nutrients that are not accessible to plant roots in return for photosynthetic carbon from plants. The carbon cost of nutrient acquisition through mycorrhizae is significantly higher than direct root uptake when soil nutrient concentration is high but becomes highly cost effective when soil nutrient supply is scarce. Raven and colleagues proposed a theoretical framework^17^ for quantifying the carbon cost of different phosphorus acquisition strategies. Each cost has two components: one being independent of soil inorganic phosphorus concentration and the other decreasing with an increase in soil inorganic phosphorus concentration. They speculated that a mixture of different strategies may be the optimal strategy of phosphorus acquisition in the field because of the large variation of inorganic phosphorus concentration and highly heterogeneous soil environment in soils. Their framework offers a way forward to assess the relative advantage or disadvantage of different nutrient acquisition strategies in a given environment and can potentially be implemented into global land models.

***Phosphorus*.** Phosphorus can limit plant growth, even though the annual requirement by plants is very small compared to the amount of soil phosphorus (Figure 1). This limitation originates partly from the uneven distribution of soil phosphorus in space and, more importantly, from most soil inorganic phosphorus being chemically or physically stabilized and unavailable for direct biological uptake. The representation of different soil phosphorus forms in land biogeochemical models is often incomplete^5^ and is usually based on limited field observations with the values of some model parameters being chosen arbitrarily. As a result, values of the same parameters can vary from model to model^18^.

Realistic representation of different soil phosphorus fractions and their dynamics is critically important for modelling phosphorus cycle on land. However, their representation in global land models remains crude^7^. Recent advances in data compilations, i.e. of soil phosphorus fractionation data^19^ and isotopic labelling experiments^20^, provide the opportunity to build data-driven models of soil phosphorus transformation. Analysis by Helfenstein *et al*.^18^ found that turnover rate of the same inorganic phosphorus pools as quantified using the Hedley fractionation varied by several orders of magnitude, which contradicted the constant rates assumed for inorganic phosphorus pools in all global land models^13–15^. The expected impact of data-constrained model parameters is a more robust quantification of the bio-availability of soil phosphorus for plant productivity and ecosystem carbon storage of natural ecosystems. This is a major source of uncertainty regarding phosphorus constraints on future land carbon uptake^4^.

Biochemical mineralization is an important process for recycling of organic soil phosphorus and is often modelled as a function of maximum biochemical mineralization rate that is assigned a constant value for a given soil type or plant functional type in global land models. While limited data are available on the activity of the soil enzymes mediating this reaction (phosphatase)^21^ and large-scale environmental drivers have been identified^21,22^, the relationship between maximum biochemical mineralization rate and enzyme activity has not yet been quantified for most soils or ecosystem types. Uses of calibrated values for maximum biochemical mineralization rate will probably remain a viable option for global modelling until more data or data synthesis becomes available.

## Major gaps

Biogeochemical models including nutrient cycles started to become part of earth system models used for climate projections about a decade ago. While significant progress has been made in model development, some major processes and fluxes remain unrepresented in current models. Here we have selected four processes as examples that likely affect nutrient cycling significantly but are currently not adequately represented in most global land models.

### Fires

Fires can affect ecosystem nutrient cycling directly through altering the physical and chemical properties of soils and by emission of nutrients from the combusted biomass and indirectly through their impact on ecosystem structure and composition. The effects of fires on nutrient cycling are well documented for many ecosystems^23,24^. However, we lack quantitative understanding of how some key processes, such as nutrient mineralization and soil microbial community biomass and composition, are affected by the intensity and frequency of fires and how those impacts vary among different ecosystems. As a result, impacts of fires on nutrient cycling are not included in most land nutrient models.

### Particulate nutrient losses by erosion

The loss of particulate matter due to soil erosion is currently omitted in land surface models, despite being the major loss pathway of phosphorus for a wide range of land ecosystems with a strong acceleration due to human land use. Estimates for natural ecosystems are scarce and highly uncertain. For global cropland area, estimates range from 1 to 26 Tg/year^7^. Representations of the transport and deposition of particulate organic matter by water for use in land surface models are being developed but have not been adapted for nutrient cycles nor included in earth system models^25^.** The inclusion of these processes in nutrient models will likely affect the simulated evolution of phosphorus fluxes in the recent past and for future scenarios due to land uses and management.

### Unresolved land surface heterogeneity

For modelling terrestrial nutrient cycles at a global scale, the use of relatively coarse spatial resolution is necessary because of limited computing resources and lack of input data to the land biogeochemical models. One consequence is that the unresolved fine-scale variations of some key processes can have significant influence on the modelled processes at coarse scales. For example, vertical or micro-scale heterogeneity can have significant impact on the simulated soil carbon profile^26,27^ and respiration^28^. Strategies for resolving land surface heterogeneity in global models are emerging^29^. One of the strategies is to develop broad-scale relationships using fine-scale process-based modelling. For example, a generic model of soil aeration developed by Yan and colleagues^30^ based on sound theoretical understanding of soil micro-scale processes provides a parameterization compatible for use in earth system models.

### Unresolved legacy effects of past disturbances

A common assumption in climate change studies is that the biogeochemical cycles are in a steady state with preindustrial boundary conditions, i.e. a state in which the stocks of matter do not change over time. However, natural and anthropogenic disturbances can push ecosystems temporarily out of equilibrium. Therefore, a steady state assumption for initial pool size can significantly affect model simulations, but such effects are rarely assessed. In the case of phosphorus where soil phosphorus is controlled by processes operating on geological time scales, use of the steady state assumption is worrisome in particular. Some model approaches use reconstructions of the observed (i.e. transient) soil phosphorus stocks to initialize soil phosphorus pools; the accuracy of these approaches depends on the representativeness of the reconstructed phosphorus pools^31^. Errors are likely to be very large for many regions where few data were used in the reconstruction. The impact of these uncertainties on simulated phosphorus cycling has not been assessed systematically.

## Outlook

Progress in modelling nutrient cycling and interlinkages with the carbon cycle in the past has been rather slow, and potentially important nutrients (like potassium) as well as key processes of the included nutrient cycles remain unresolved in models. While future efforts are required to include key missing processes and explore additional nutrients, one major difficulty in modelling nutrient cycles at a global scale is the lack of ready-to-use data for model development and evaluation. On the other hand, a large number of scattered small-scale field observations has not been used for developing or evaluating land biogeochemical models. In the following, we outline two complementary approaches aiming at bridging the gap between models and data.

### Data mining using machine-learning

Lack of reliable global data has been a major obstacle in developing land nutrient models, e.g. for phosphorus. Furthermore, the definitions of different functional pools of soil phosphorus vary significantly among different models. As a result, the predicted stock of labile soil phosphorus for a tropical forest in the Amazon varied by a factor of 10 among the models that were driven by the same boundary conditions^4^. Recently, the number of measurements of soil phosphorus has significantly increased^19^: for example, vertical profiles of soil phosphorus are available from over 2,000 sites in Australia and China alone, but they have only limited use for global modelling because the area as represented by those field measurements is much smaller than the spatial resolution of a typical global model (from 50 by 50 km^2^ to 200 by 200 km^2^).

Machine learning may offer a solution^32^ to bridge the gap between scales. Machine learning can be used to develop relationships between climate, soil, and vegetation and the target variables, such as total soil phosphorus and its fractions, using site-scale observations in different regions or globally. Those relationships can then be used to upscale the scattered site-level observations to a global scale. The resulting large-scale dataset can be used to benchmark soil phosphorus models and to identify environmental drivers. Recently, Sun *et al*.^22^ applied this approach to a compilation of measurements of phosphatase activity, which governs the recycling of soil phosphorus. Their approach was limited to Europe because of the lack of data available elsewhere, which indicates that data availability remains a major bottleneck. With increasing field observations^33^ and applications of advanced machine learning, we will develop a better understanding of plant–microbe interaction and nutrient cycles at a regional to global scale.

### Optimality principles

Basic ecological principles based on optimization, such as the evolutionary stable strategies, maximizing fitness, or minimizing the cost of risks, offer an independent constraint on the predictions made by biogeochemical models^34^. This is particularly useful for the global land models of nutrient cycles with many poorly understood processes.

An example is the representation of nutrient acquisition by plants. Current approaches to model nutrient uptake vary from taking the minimum of nutrient demand and soil supply to representing the competition among multiple soil organisms and plant roots for available nutrients^14^. By applying optimality principles, Lu and Hedin^35^ successfully predicted a global pattern of plant symbiotic relationships with soil microbes as the observed^36^ with a much lower number of parameters than most conventional biochemical models. While it may not be possible to apply optimality principles to global land models directly, it is possible to apply them first in simplified land models as a transitional step. Results from these studies may help improve the representation of nutrient acquisition in modelling nutrient cycles in the future.

However, there are some important caveats of optimality principles. One is the definition of the optimum. For example, an optimal nutrient acquisition that maximizes the marginal return per carbon investment depends on cost and benefit, and that optimal strategy can be very different from the one based on an evolutionary stable strategy that accounts for competition and life history. What implications those two different optima have on the modelled nutrient cycle is yet to be explored. Answers to this question may guide the development of models of nutrient acquisition strategy for different plant functional types in the future.

### The combined approach

The above two approaches are complementary. Data mining can be used to identify key patterns within a large number of observations but does not provide insights into specific processes as represented in the global models, while the optimality approach can be used to quantify those key processes in winning the competition. That winning strategy is critically dependent on how cost and benefit are constructed, which would be guided by the patterns as identified from data mining. Together, these two approaches can be powerful in studying some partially understood systems, such as global nutrient cycles on land.
